# Distal interphalangeal joint extensor tendon enthesopathy in patients with nail psoriasis

**DOI:** 10.1038/s41598-019-39985-7

**Published:** 2019-03-06

**Authors:** Magdalena Krajewska-Włodarczyk, Agnieszka Owczarczyk-Saczonek, Waldemar Placek, Maja Wojtkiewicz, Andrzej Wiktorowicz, Joanna Wojtkiewicz

**Affiliations:** 1 Department of Rheumatology, Municipal Hospital in Olsztyn, 10-900 Olsztyn, Poland; 2 Department of Internal Medicine, School of Medicine, Collegium Medicum, University of Warmia and Mazury, 10-900 Olsztyn, Poland; 3 Department of Pathophysiology, School of Medicine, Collegium Medicum, University of Warmia and Mazury, 10-900 Olsztyn, Poland; 4 Department of Dermatology, Sexually Transmitted Diseases and Clinical Immunology, School of Medicine, Collegium Medicum, University of Warmia and Mazury, 10-900 Olsztyn, Poland; 5 Faculty of Earth Sciences, Department of Geomatics and Cartography Nicolaus Copernicus University, Torun, Poland; 6 DRAMIŃSKI S.A. Ultrasound Scanners, Olsztyn, Poland; 7 Laboratory for Regenerative Medicine, School of Medicine, Collegium Medicum, University of Warmia and Mazury, 10-900, Olsztyn, Poland

## Abstract

The aim of the study was an ultrasound assessment of distal interphalangeal (DIP) joint enthesopathy in patients with nail psoriasis. Altogether, 72 patients with nail psoriasis (41 with psoriasis and 31 with psoriatic arthritis) and 30 people in the control group participated in the study. In total, 1014 nails were examined. The thickness of DIP digital extensor tendons in the groups of patients with psoriasis (Ps) and psoriatic arthritis (PsA) was correlated with the nail bed thickness (r = 0.316, p = 0.027 vs. r = 0.402, p = 0.031, respectively) and with the thickness of the nail matrix in patients with psoriasis (r = 0.421, p = 0.012). The linear regression model showed the tendon thickness in Ps patients to be affected by the nail bed thickness, duration of psoriasis and the thickness of the nail matrix, whereas in PsA patients it was found to be significantly affected by duration of psoriasis and of arthritis, the nail bed thickness, CRP concentration and the swollen joint count. Our findings may indicate the role of the nail-tendon apparatus changes in the PsA development and they emphasise the justifiability of US examinations in patients with psoriasis direct assessment of morphological changes in nails as potential predictors of PsA development.

## Introduction

Nail psoriasis is one of clinical manifestations of psoriasis (Ps); it is a typical, disfiguring picture of local changes and is associated with pain and functional disorders. Moreover, involvement of nails is one of the known risk factors for the development of psoriatic arthritis (PsA)^1^, which especially affects distal interphalangeal joints (DIP)^2^. Psoriatic changes in nails are present in approx. 10–55% of patients with psoriasis, but the risk of the occurrence of such changes during a patient’s lifetime may be as high as 80–90%^3^. Because of anatomical factors, inflammation associated with nail psoriasis may spread to adjacent structures, including DIP joints and digital extensor tendons^4^. Enthesopathies are known to be among the characteristic features of psoriatic arthritis^5,6^. An assessment of psoriatic changes in nails is practically based on clinical assessment indices, such as nail psoriasis severity index (NAPSI) and modified NAPSI (mNAPSI)^7,8^. Useful imaging methods include ultrasound (US) examination^9^ and magnetic resonance imaging (MRI), so far being the main methods in imaging of articular changes^10^, and optical coherence tomography^11^. Being a non-invasive and a relatively cheap method, US seems to be promising for use in assessment of progression of changes in nails and adjacent structure as well as treatment outcome. An early diagnosis of psoriatic arthritis or enthesopathies may significantly affect the therapeutic decisions and prognosis.

## Aim of the Study

The aim of the study was to conduct an ultrasound assessment of distal interphalangeal joint enthesopathy in patients with nail psoriasis without arthritis compared with patients with PsA and a control group and an assessment of an association between enthesopathy and selected clinical factors.

## Materials and Methods

Altogether 102 patients participated in the study, including 72 successively registered patients with psoriasis with nail involvement (41 with psoriasis and 31 with psoriatic arthritis), treated at the Dermatology Clinic, at the Rheumatology Clinic and at the Department of Rheumatology of the Municipal Hospital in Olsztyn and at the Clinic of Dermatology, STD and Clinical Immunology at the University of Warmia and Mazury, and 30 people without psoriasis or psoriatic arthritis. Psoriatic arthritis were diagnosed based on CASPAR criteria^12^. The subjects’ age ranged from 30 to 64 years.

All of the patients were examined by an experienced dermatologist. The macroscopic progression of psoriatic changes in nails with pitting, with hyperkeratosis and/or onycholysis was assessed by mNAPSI. The intensity of psoriatic changes in the skin was assessed with the PASI (Psoriasis Area and Severity Index)^13^.

The activity of psoriatic arthritis was assessed by the Disease Activity Score calculated for 28 joints (DAS 28)^14^ and the number of tender (tender joint count – TJC) and swollen (swollen joint count – SJC) joints, calculated from 68 and 66 joints, respectively.

A US examination of nails and DIP joint extensor tendons was conducted in all the patients and people in the control group. The examination was conducted by a single rheumatologist experienced in ultrasound examinations of the musculoskeletal system. Morphological changes were examined with DermaMed equipment and software (DRAMIŃSKI, Olsztyn, Poland) with a linear head with frequency ranging from 12 to 48 MHz. All the nail examinations (1,014 nails, 6 nails were excluded because of a previous injury) were conducted at 24 MHz. An assessment of intensified blood supply, corresponding to intensification of inflammation, was made with a Mindrey M5 (Mindray, Guangdong, China) apparatus with the Power Doppler (PD) technique. An assessment of the nails, extensor tendons and DIP joints was made by placing the head on the dorsal side. In order to avoid pressure on surface tissues, an appropriate amount of gel without gel pads was used. In order to avoid artefacts, the intensified flow, visible in the PD technique was confirmed by pulsed wave Doppler spectrum. The nail thickness was measured as the maximum distance between the dorsal and ventral nail plates. The nail bed thickness was measured as the distance between the ventral plate and the bone margin of the distal phalanx. The nail matrix thickness was measured at the proximal end of the nail bed.

According to the classification proposed by Wortsman *et al*.^15^, morphological changes in nails in US images were described as: focal hyperechoic involvement of the ventral plate (type I), loosening of the borders of the ventral plate (type II), wavy plates (type III) and loss of definition of both plates (type IV).

Tendon attachments were assessed in accordance with OMERACT (Outcome Measures in Rheumatology)^16^ recommendations in a US examination, in the scale of greyness, at the place where an extensor tendon is attached to the distal phalanx of the DIP. Loss of normal fibrillary architecture, thickened tendon or enthesophytes at its bony insertion and bony changes including erosions were regarded as enthesopathies. Inflammation-associated increased vascularisation was assessed with the PD and verified by pulsed wave Doppler spectrum. The tendon thickness was measured at the site where it is attached to the distal phalanx.

Inflammatory markers were measured with two standard laboratory parameters: erythrocyte sedimentation rate assessed using BD Vacutainer Sedi-15 equipment (BD, Franklin Lakes, NJ, USA) and concentration of C-reactive protein measured with a standard immunoturbidimetric method using a COBAS 6000 INTEGRA apparatus (Roche Diagnostics, Mannheim, Germany).

Patients with visible DIP joint deformations or diagnosed with osteoarthritis of hands and with changes in nails other than caused by psoriasis were excluded from the study. People who do hard physical labour were also excluded from the study in order to eliminate from the assessment any changes in tendons caused by overload.

Informed consent from all subjects was obtained prior to their participation. All the patients gave written consent to publish the results. The experiment was approved by the Bioethics Committee at the Warmia and Mazury Chamber of Physicians (OIL 625/16/Bioet; 21.12.2016). This study adhered to the recommendations outlined in the Declaration of Helsinki Principles.

## Statistical Analysis

StatSoft program, Inc. STATISTICA, version 12.5 (StatSoft, Tulsa, OK, USA) was used for calculations. Obtained results were presented as an arithmetic mean and standard deviation, medians and interquartile ranges or frequencies (percentages). The χ^2^ test, the Mann-Whitney U-test, the Kruskal-Wallis test and Student’s t-test were performed as appropriate. The presence of the relationship between quantitative features was tested using Pearson’s correlation coefficient for parameters consistent with normal distribution and Spearman’s correlation coefficient in the case of non-compliance with normal distribution. To evaluate the relationship between the studied data, linear regression modelling was used. Variable models were selected stepwise using backward elimination. The statistical level of significance was p < 0.05.

## Results

In total, 102 people aged 30–64 years participated in the study, including 72 patients with nail psoriasis (41 with psoriasis without arthritis and 31 with psoriatic arthritis) and 30 people in the control group. There was no difference between the groups regarding the age or sex. Greater intensity of dermal changes compared to PsA patients was observed in patients with psoriasis, whereas significantly higher inflammation parameters were observed in patients with arthritis. No differences were observed in both groups in the intensity of changes in nails as assessed with the mNAPSI. The duration of psoriasis in both the groups of patients did not differ significantly (Table 1).

**Table 1:** Age and clinical characteristics of the patients studied.

	Ps (n = 41)	PsA (n = 31)	Control (n = 30)	p
male/female (number)	22/19	15/16	14/16	—
Age (years, mean ± SD)	47.6 ± 9.6	49.7 ± 10.8	49.4 ± 12.7	ns
Ps duration (years, median, IQR)	17.5 ± 11.3	19.3 ± 6.6	—	ns
PsA duration (years, median, IQR)	—	7.8 ± 7.1	—	—
DAS 28 (mean ± SD)	—	3.3 ± 0.6	—	—
PASI (mean ± SD)	6.4 ± 3.6	4.7 ± 3.7	—	0.034
mNAPSI (mean ± SD)	21.8 ± 16.2	20.3 ± 15.2	—	ns
ESR (mean ± SD)	13.5 ± 6.8	25.7 ± 10.2	—	0.017
CRP (mg/dl, mean ± SD)	2.4 ± 2.5	9.4 ± 4.1	—	<0.001
TJC (number, mean ± SD)	—	2.8 ± 1.4	—	—
SJC (number, mean ± SD)	—	2.3 ± 0.6	—	—

Results are presented as numbers, mean values and standard deviations (SD) or medians and interquartile ranges (IQR).

Ps: psoriasis, PsA: psoriatic arthritis, DAS: disease activity score, PASI: psoriasis area severity index, mNAPSI: modified nail psoriasis severity index; ESR: erythrocyte sedimentation rate, CRP: C-reactive protein, TJC: tender joint count; SJC: swollen joint count.

**Figure 1:**
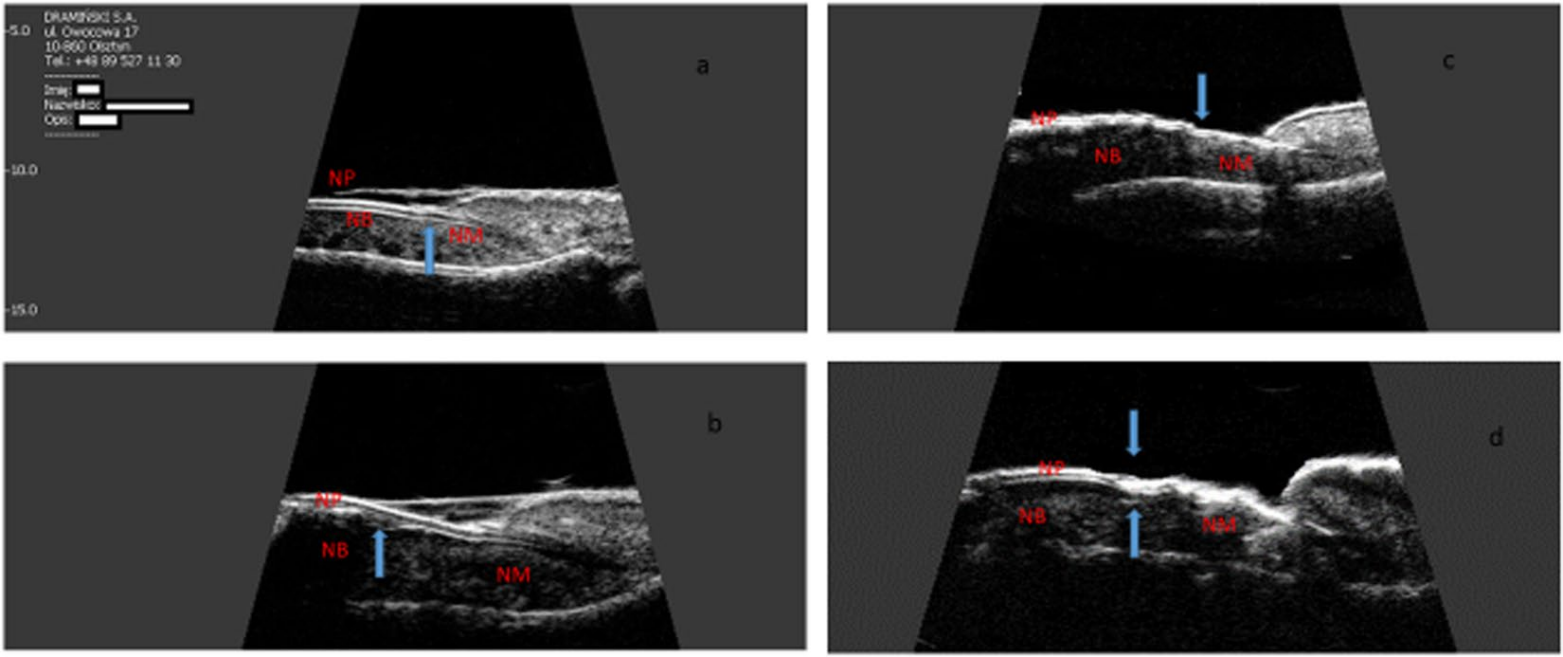
Longitudinal scan of psoriatic nail. (**a**) Focal hyperechoic involvement of the ventral plate. (**b**) Loosening of the borders of the ventral plate. (**c**) Wavy plates. (**d**) Loss of definitione of both plates. NP: nail plate; NB: nail bed; NM: nail matrix. Sonographic image obtained by MK-W.

A total of 1014 nails were examined: 406 nails in patients with psoriasis, 310 nails in patients with PsA and 298 nails in people in the control group. Psoriatic changes in patients with Ps and PsA were present in 272 (67%) and 168 (54%) nails, respectively. The US examination of the affected nails revealed an increased thickness of the nail plate, bed and matrix in both groups of patients compared to the control group. The thickness of digital extensor tendons in both patients groups was significantly greater than in the control group (Table 2). The thickness of extensor tendons in patients with PsA was also greater than in patients with psoriasis without arthritis (p = 0.022). The nails with psoriatic changes were assessed in a US examination in regard to their morphology, in accordance with the classification proposed by Wortsman *et al*. (Fig. 1). Focal hyperechoic involvement of the ventral plate (type I), loosening of the borders of the ventral plate (type II), wavy plates (type III) in patients with psoriasis was observed in 86.5%, 10% and 3.5% of the nails under examination, respectively. No loss of definition of both plates (type IV) was observed. Type I, II, III and IV changes were present, respectively, in 16%, 77%, 5% and 2% of the patients with PsA (Table 3). The digital extensor tendon was the thickest in type II of the changes in US in patients with Ps (p = 0.023). No difference in the thickness of the extensor tendon in type II, III or IV changes was observed in the group of patients with arthritis. The tendon thickness differed significantly between type I and the other types with involvement of the inner nail plate (p = 0.011). After the patients with Ps and PsA were grouped according to clinical manifestations of psoriatic changes in nails, the thickness of the digital extensor tendon did not differ in the onycholysis and hyperkeratosis type changes (concomitant or occurring separately) in both groups and it was significantly greater than in the pitting type of changes (p = 0.043 and p = 0.027, respectively).

**Table 2:** US measurements of the fingers with psoriatic nail involvement compared to a control.

	Ps (272/406)	PsA (168/310)	Control (n = 298)	p
NP thickness (mm)	0.74 ± 0.14	0.72 ± 0.08	0.51 ± 0.12	<0.001
NB thickness (mm)	2.02 ± 0.39	2.03 ± 0.34	1.76 ± 0.22	0.003
Matrix thickness (mm)	1.97 ± 0.35	2.01 ± 0.24	1.89 ± 0.27	0.031
Tendon thickness (mm)	0.97 ± 0.14	1.10 ± 0.21	0.89 ± 0.11	<0.001

Results are presented as mean values and standard deviations (SD).

NP: nail plate; NB: nail bed;

**Table 3:** Wortsman classification of the psoriatic nails studied.

Wortsman classification	Ps (n = 302)	PsA (n = 168)
I	231	27
II	31	129
III	10	9
IV	—	3

**Figure 2:**
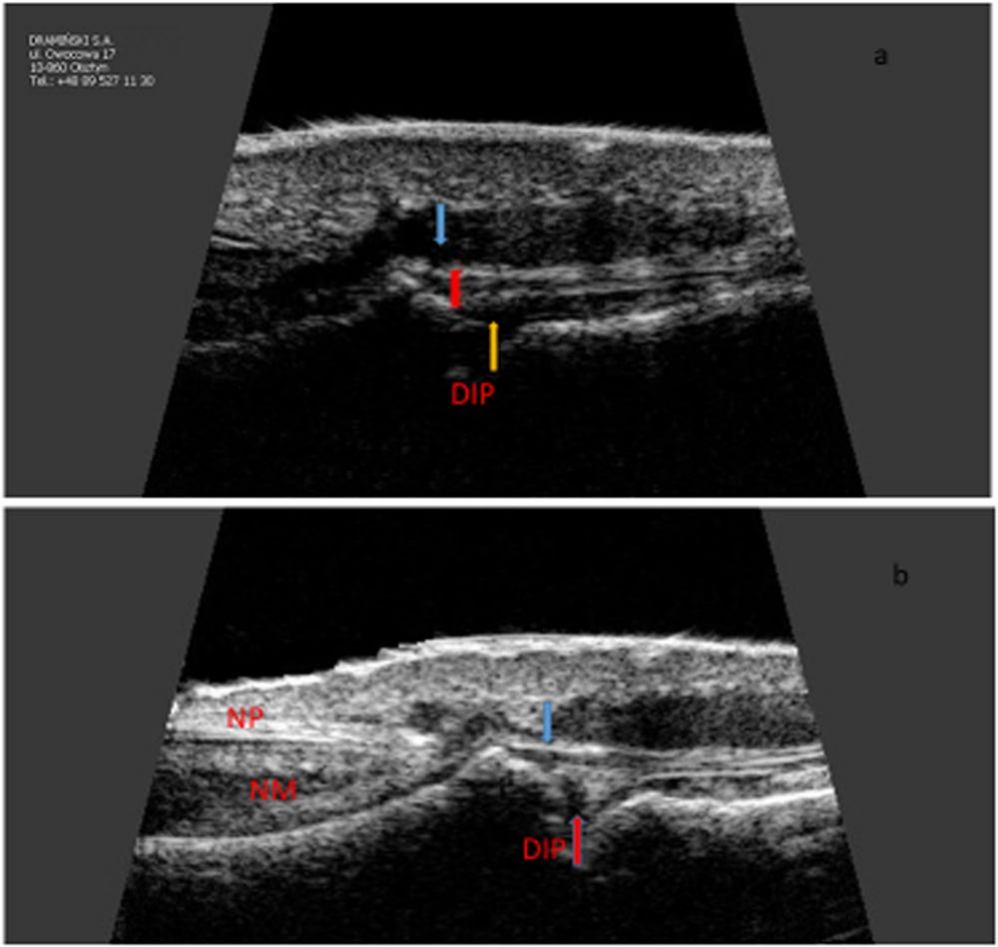
Longitudinal scan of DIP and finger extensor tendon in psoriatic arthritis. (**a**) Extensor tendon calcification (blue arrow), thickeness of the finger extensor tendon (red line), osteophyte (yellow arrow). (**b**) Bony erosion (blue arrow), loss of normal fibrillary architecture of extensor tendon. DIP: distal interphalangeal joint; NP: nail plate; NM: nail matrix. Sonographic image obtained by MK-W.

On the greyness scale, in the US examination of tendon attachments of digital extensors with clinical involvement of nails and with no changes in nails in the groups of patients with Ps and PsA, changes in the type of loss of normal fibrillary architecture, enthesophytes and bony changes including erosions were observed more frequently in DIP joints with nail involvement (Fig. 2). In patients with Ps, loss of normal fibrillary architecture, enthesophytes and bony changes were observed in 84/272 (31%) vs. 25/130 (19%), 40/272 (15%) vs. 12/130 (9%) and 24/272 (9%) vs. 7/130 (5%) DIP joints with and without changes in nails. In patients with Ps, enthesopathies were present more frequently in digits with nail involvement than in those in which nails were not affected (104/272 (38%) vs. 34/130 (26%), respectively, p = 0.047). Increased vascularisation as assessed by the PD technique within the tendon attachments under study in Ps was observed in 69/272 (25%) digits with psoriatic nails and in 19/130 (15%) digits with no clinical changes in the nails (p = 0.043). In patients with PsA, loss of normal fibrillary architecture, enthesophytes and bony changes were observed in 87/168 (52%) vs. 63/142 (44%), 46/168 (27%) vs. 24/142 (17%) and 34/168 (20%) vs. 16/142 (11%) DIP joints of the digits under examination with and without involvement of the nails. In patients with PsA, enthesopathies were present in 114/168 (68%) digits with nail involvement and in 80/142 (56%) digits with no changes in the nails; the differences were not statistically significant. However, increased vascularisation in the area of the tendon attachments of digital extensor in DIP joints was observed more frequently when they were concomitant changes in the nails than without such changes, in 83/168 (49%) vs. 41/142 (28%) (p = 0.031). No erosions were observed in the tendon attachments under study in the control group. Loss of normal fibrillary architecture and enthesophytes were observed in this group in 17/298 (6%) and 14/298 (5%) digits, respectively.

The intensity of dermal changes in all patients did not affect the thickness of the digital extensor tendon in a DIP joint. Among the patients with Ps and PsA, the tendon thickness increased with the duration of psoriasis (r = 0.311, p = 0.039 vs. r = 0.223, p = 0.014, respectively). Among the subjects with PsA, the tendon thickness also increased with the duration of arthritis (r = 0.469, p = 0.029). In the group with arthritis, an increase in the thickness of the digital extensor tendon was correlated with the swollen joint count (r = 0.301, p = 0.044) and CRP concentration (r = 0.268, p = 0.038) and the ESR increase (r = 0.521, p = 0.041), but it was not correlated with the tender joint count or the disease activity as measured by the DAS28. The thickness of digital extensor in the DIP joint in the groups of patients with Ps and PsA was correlated with the nail bed thickness (r = 0.316, p = 0.027 vs. r = 0.402, p = 0.031, respectively), but not with the nail plate thickness. The thickness of the tendon under study in patients with psoriasis was also correlated with the nail matrix thickness (r = 0.421, p = 0.012) (Table 4). The thickness of the digital extensor in the control group increased with the patients’ age (r = 0.218, p = 0.046). No relationship was observed in this case between the tendon thickness and the thickness of the nail matrix, bed or plate.

**Table 4:** Correlations between the finger extensor tendon thickness and the thickness of the nail structures studied.

	NB thickness	Matrix thickness	NP thickness
finger extensor tendon thickness in Ps (n = 406) r, *(p)*	0.316	0.421	0.071
	*−0.027*	*−0.012*	*−0.241*
finger extensor tendon thickness in PsA (n = 310) r, *(p)*	0.402	0.141	0.146
	*−0.031*	*−0.093*	*−0.109*

Ps: psoriasis; PsA: psoriatic arthritis, NB: nail bed, NP: nail plate.

When the linear regression model was applied which consisted in taking into account in the original model of all potential variables, selected stepwise with backward elimination, the factors were determined which, in combination, had the greatest effect on the thickness of the digital extensor tendon in the DIP joint in the patients under examination. The tendon thickness in psoriatic patients with no arthritis was found to be affected by the thickness of the nail bed, duration of the skin disease and the nail matrix thickness (Table 5). The tendon thickness in the group of PsA patients was found to be significantly affected by duration of psoriasis and of arthritis, the nail bed thickness, CRP concentration and the swollen joints count (Table 5).

**Table 5:** The regression coefficients in modelling for finger extensor tendon thickness in psoriasis and psoriatic arthritis.

	Ps (fingers, n = 406), Adjusted *R*^2^: 0.367	PsA (fingers, n = 310), Adjusted *R*^2^: 0.303
Age	0.1657	0.1998
	−(0.142)	−(0.159)
Ps duration	0.4406	0.4921
	−(0.001)	(<0.001)
PsA duration	—	0.4104
		(<0.001)
NB thickness	0.4842	0.391
	(<0.001)	(<0.001)
Matrix thickness	0.4021	0.1172
	−(0.017)	−(0.106)
NP thickness	0.2011	0.1801
	−(0.089)	−(0.105)
CRP	0.1816	0.3641
	−(0.086)	−(0.002)
ESR	0.1758	
	−(0.127)	
DAS 28	—	0.1674
		−(0.096)
SJC	—	0.3363
		−(0.006)
TJC	—	0.0207
		−(0.099)

*The p-value is shown in brackets.

Ps: psoriasis; PsA: psoriatic arthritis; NB: nail bed; NP: nail plate, CRP: C-reactive protein, ESR: erythrocyte sedimentation rate, DAS 28: disease activity score 28 joints, SJC: swollen joint count, TJC: tender joint count.

In total, enthesopathies of at least one DIP joint, including loss of normal fibrillary architecture, thickened tendon or enthesophytes and bony changes, occurred more frequently in patients with psoriatic arthritis (PsA) than in those with psoriasis (Ps); they were present in 16 (52%) and 14 (34%) of the patients, respectively. Such changes affected only 4 (13%) people in the control group.

## Discussion

Enthesopathies are one of extra-articular manifestations of spondyloarthropathies. Enthesitis is more common in PsA than other forms of arthritis including rheumatoid arthritis and ankylosing spondylitis^17^ and has been reported to occur in 35–50% in PsA. Enthesitis is associated with adjacent osteitis or bone and synovial inflammation and damage in the peripheral and axial joints and is considered to be involved in the pathogenesis of PsA^18^. Recently, it was proposed that autoinflammatory reaction at predisposed sites leading to an innate immune response is the triggering mechanism for PsA^6^. Enthesitis may be asymptomatic especially in the early stages of PsA and psoriasis without arthritis^19^. Anatomically, there is strong link between PsA and nail inflammation. Psoriatic changes in nails are a known risk factor for the development of arthritis^20,21^.

The features of enthesopathies of the digital extensor tendon in the DIP joint, as determined in the US examination, were present in 34% patients with psoriasis with nail involvement without the concomitant arthritis and in 52% of PsA patients. The features of enthesopathies were observed in joints of digits with nail involvement both in patients with Ps and with PsA (38% vs. 66%, respectively). The thickness of the extensor tendon at the attachment to the distal phalanx was found to increase significantly in both groups in cases when the inner nail plate was affected. The literature provides reports on more frequent occurrence of enthesopathies in psoriasis without arthritis^22,23^, but only several studies have been conducted so far of using US to assess the connection between psoriatic changes in nails with enthesopathy of the digital extensor tendon in the DIP joint. In a recently published paper by Acosta-Felquer *et al*., the frequency of enthesopathies in patients with psoriasis and PsA in a US examination of digits with nail involvement did not differ and was 61% and 60%, respectively^24^. In another study, Ash *et al*. described the relationship between the occurrence of psoriatic changes in nails and the intensity of enthesopathies in joints other than digital^25^ suggesting that enthesitis may be not only a focal response.

The thickness of the digital extensor tendon in the DIP joint was much greater when nails were affected in both groups of patients examined in this study. Moreover, the tendon thickness increased more when onycholysis and/or hyperkeratosis was present than when pitting-type changes occurred. Aydin *et al*. presented the findings of a study in which onycholysis and pitting were the most frequent changes in nails in digits with increased thickness of the extensor tendon in the DIP joint^26^. In another study, Castellanos-González *et al*. conducted US examinations in patients with Ps and found the presence of enthesopathy in 31% of patients with onycholysis. The frequency of changes in the digital extensor tendon or its attachment in the DIP joint with nail involvement was nearly 83%^27^.

In both groups of patients, morphological changes in nails by US examination were assessed in accordance with the classification proposed by Wortsman *et al*. Type I (focal hyperechoic of ventral plate) was usually found in an examination of the affected nails in patients with psoriasis without arthritis. The ultrasound changes in the affected nails in patients with PsA usually involved loosening of the borders of the ventral plate (type II). Similar findings were reported by Sandobal *et al*.; in their study, type I was found in 16/20 of the Ps patients, whereas type II was found in 34/35 of the PsA patients, irrespective of the clinical manifestations of psoriatic nail involvement^28^. Apart from nail plates, in our study an ultrasound assessment of the nail bed and matrix was conducted. The nail bed thickness was correlated with the thickness of the digital extensor tendon. Aydin *et al*. did not find such a correlation, but the thickness of the digital extensor tendon as measured in their study was correlated with the skin thickness as assessed above the DIP joint^26^.

Increased vascularisation may be one of the manifestations of an open or subclinical inflammation. As expected, increased vascularisation around the digital extensor tendon in the DIP joint, as revealed by the PD technique, was observed in the PsA patients more frequently than in those with Ps. In addition, an intense PD signal was observed around the tendon in the digital DIP joints with nail involvement in both groups of patients. Unlike in the study by Acquiter *et al*., no difference regarding the PD signal in DIP joints was observed in Ps patients between those with and without affected nails^29^. Sandobal *et al*. observed increased vascularisation in 106/350 DIP joints in PsA patients and only in 8/200 joints in Ps patients, irrespective of changes in nails^28^. The study by Acosta-Felquer *et al*. showed the frequency of a PD signal in the extensor tendon and the DIP joint in groups of Ps and PsA patients to be higher when nails were affected, but there was no difference between patients with and without arthritis^24^.

Some limitations to our study must be mentioned. The first limitation was a consequence of the fact that the changes in nails could not be hidden during the US examinations. This prevented the full blinding of the study. Second, all US were performed by the same experienced rheumatologist with no intra-observers agreement analysis performed. Limitations of the study also include the cross-sectional design. For future studies, a longitudinal design would allow the assessment of predictors of distal interphalangeal joint extensor tendon enthesitis.

## Conclusions

The presence of psoriatic changes in the nail in the Ps and PsA patients under study was associated with changes in US examinations in the digital extensor tendon in the DIP joint. The thickness of the digital extensor tendon in patients with nail psoriasis without arthritis was found to be affected by the thickness of the nail matrix and bed and duration of the skin disease. In the PsA patients it was found to be affected by duration of the skin and joint disease, the nail bed thickness, CRP concentration and the swollen joints count. The usability of US examinations in nail psoriasis must be confirmed in further studies.

## Data Availability

The datasets generated during and/or analysed during the current study are available from the corresponding author on reasonable request.
